# Supplementary material for: Intensified visual clutter induces increased sympathetic signalling, poorer postural control, and faster torsional eye movements during visual rotation
Source: PLoS One. 2020 Jan 3;15(1):e0227370. doi: 10.1371/journal.pone.0227370 (PMC6941927; doi:10.1371/journal.pone.0227370)
Supplement: S1 Data — (PDF) [file pone.0227370.s001.pdf]

No	Sex	Intensity order	Direction	Age	T1 s05 CTv	T2 s05 CTv	T3 s05 CTv	T1 s1 CTv
1	0	1	0	0	2,595238	2,533333	0,714286	1,972222
2	0	0	0	0	4,3125	2,3125	1,772727	3,846154
3	0	1	1	0	1,04	0,826087	0,575758	0,388889
4	1	0	1	0	4,25	5,818182	1,9	4
5	1	1	0	0	1,310811	1,5	1,536585	3,208333
6	1	0	0	0	0,682927	0,864198	0,642857	2,263158
7	1	1	1	0	4,941176	3,615385	4,714286	6,375
8	0	0	1	0	2,735294	1,433333	1,782609	3,195652
9	0	1	0	1	4,25	3,872093	0,685185	3
10	0	0	0	1	1,842105	1,481481	1,6	2,35
11	0	1	1	1	1,411765	1,8	1,105263	3,384615
12	0	0	1	1	0,647059	1,086957	1,090909	1,576923
13	1	1	0	1	2,411765	0,636364	0,897059	2,9
14	1	0	0	1	0,9	2,166667	0,857143	2,068182
15	1	1	1	1	1,973684	1,140625	1,092593	3,304348
16	1	0	1	1	6,272727	2,5	2,333333	7

Low intensity	s05
High intensity	s1
Body-sway	BS
Pupil size	PS
Time periods	T1-3
Amplitude	Amp
Frequency	Hz
CT	Cyclotorsion
VV	Vertical Vergence
Intensity Order	0= LI first
Direction	0= Clockwise
Age	0=young
Sex	0= male

T2 s1CTv	T3 s1 CTv	AverageTs(	AverageTs:	T1 s05 VVv	T2 s05 VVv	T3 s05 VVv	T1 s1 VVv	T2 s1VVv	T3 s1 VVv
1	0,967742	1,947619	1,313321	0,368039	1,379692	0,896343	0,999737	0,810911	0,330035
4,583333	2,72449	2,799242	3,717992	1,601886	1,442362	0,877407	1,264367	1,292479	0,781007
0,928571	0,961538	0,813948	0,759666	0,55799	0,396728	0,537146	0,621462	0,394012	0,734014
4,666667	5,057143	3,989394	4,574603	1,463833	0,490684	0,685746	0,652644	0,623736	0,357163
1,12	1,3	1,449132	1,876111	0,348526	0,409143	0,544197	0,669872	0,920938	0,907642
1,357143	1,666667	0,729994	1,762322	0,810982	0,168437	0,834325	0,82413	0,270353	0,489216
4,4	2,8	4,423616	4,525	1,104606	0,769152	1,617341	2,668135	1,085138	0,985513
2,357143	2,029412	1,983745	2,527402	0,886081	1,321136	0,691716	0,757843	0,545664	1,069385
3,571429	1,423077	2,935759	2,664835	1,055237	0,540079	0,627657	1,278547	0,708067	0,811074
3,625	1,785714	1,641196	2,586905	0,832532	0,438021	0,595431	3,66862	1,560836	1,081656
1,166667	0,848485	1,439009	1,799922	0,833265	0,869374	1,708343	0,51214	0,667348	0,738909
0,92	1,769231	0,941641	1,422051	1,059729	0,819506	0,558598	1,263703	1,431628	1,31345
1,5	0,803571	1,315062	1,734524	1,061861	0,729569	0,727765	0,972592	0,523853	0,434197
0,821429	2,0625	1,307937	1,650703	0,768839	0,703107	0,331086	0,565156	0,110919	1,145457
1,424658	0,691358	1,402301	1,806788	1,217391	0,888889	0,754179	1,571429	0,772727	0,506239
4,513158	4,823529	3,70202	5,445562	1,257143	0,493583	0,453846	1,727273	0,858952	0,333333

AverageVV	AverageVV	T1 s05 OBF	T2 s05 OBF	T3 s05 OBF	T1 s1 OBR	T2 s1 OBR	T3 s1 OBR	AverageOE	AverageOE
0,881358	0,713561	7,051528	1,836159	0,796889	1,97274	1,233182	2,932242	3,228192	2,046055
1,307218	1,112618	2,69214	1,603273	2,020416	3,041961	3,546157	3,488431	2,105276	3,35885
0,497288	0,583163	1,863831	2,082251	1,071882	0,625765	2,35671	1,309972	1,672655	1,430816
0,880088	0,544514	2,903336	11,85729	2,770705	6,128911	7,481801	14,15919	5,843776	9,256635
0,433955	0,832817	3,761015	3,666199	2,823584	4,789472	1,216152	1,432282	3,416932	2,479302
0,604581	0,5279	0,842099	5,130679	0,770512	2,746119	5,019884	3,406811	2,247763	3,724271
1,163699	1,579595	4,47325	4,70048	2,914838	2,389309	4,054782	2,841161	4,029523	3,095084
0,966311	0,790964	3,086956	1,084925	2,577083	4,216774	4,319768	1,897738	2,249654	3,478093
0,740991	0,932563	4,027529	7,169497	1,091656	2,346413	5,043916	1,754559	4,096227	3,048296
0,621995	2,103704	2,212654	3,382215	2,68713	0,640568	2,322473	1,650907	2,760667	1,537983
1,136994	0,639466	1,694256	2,070456	0,64698	6,60877	1,748212	1,148294	1,470564	3,168425
0,812611	1,33626	0,610589	1,326356	1,952943	1,247859	0,642625	1,34701	1,296629	1,079165
0,839732	0,643547	2,271262	0,872246	1,232622	2,981724	2,863397	1,850708	1,45871	2,565276
0,60101	0,607177	1,170597	3,081562	2,588885	3,659487	7,405678	1,800591	2,280348	4,288585
0,953486	0,950131	1,621241	1,283203	1,448719	2,102767	1,843674	1,365676	1,451054	1,770706
0,734857	0,973186	4,989669	5,065001	5,141243	4,052632	5,254259	14,47059	5,065304	7,925826

BSS1-befor	BS1-during	BSS1-after	BSS05-befo	BSS05-duri	BSS05-afte	PS1-before	PS1-during	PS1-after	PS05-befor
0,9359	1,03733	0,4417	1,97295	1,10081	1,61702	33,76511	32,39363	33,26663	33,16549
1,48828	0,57044	0,17379	0,842	0,74665	0,35249	44,41226	43,82981	43,18741	46,89061
0,77852	0,63629	0,35905	0,49741	0,35732	0,35165	39,29614	40,094	40,13607	39,32269
0,75283	1,2251	0,97393	1,026	0,50992	0,93878	43,84642	43,16845	44,63609	41,39863
2,70655	1,13494	2,45582	0,85307	1,16361	0,9994	39,39905	38,40006	40,33082	36,59198
0,69089	0,24862	0,42057	0,55888	0,18615	0,23807	31,20128	30,68228	31,06363	30,04465
1,28649	1,48618	1,00278	0,84851	0,98527	0,48819	41,20908	40,02276	38,87439	39,73755
1,13461	0,38235	0,71251	0,556	0,33571	0,53847	42,721	39,9777	40,30364	40,99298
1,93771	2,8933	2,80498	2,20507	1,50241	1,27831	37,59213	36,12363	38,12308	37,38812
0,57294	0,3156	0,16809	0,38165	0,49049	0,14816	30,53926	29,57662	29,57522	30,11593
2,67275	1,20051	0,45762	0,95514	1,08924	0,78168	39,78868	37,43739	38,47247	39,69001
1,02706	1,02039	1,11687	1,3856	1,07411	1,31575	31,99048	32,97936	32,91364	32,46874
0,88667	0,522	1,23939	0,74381	0,56991	0,52133	27,95214	28,01236	28,51421	27,95193
2,16448	1,3867	3,89366	2,93438	1,04492	0,38332	31,07856	29,97373	30,16579	31,65196
1,8579	0,51045	0,93416	0,80217	0,67213	0,41423	31,99534	30,96753	30,59626	28,27759
1,00666	2,41886	0,39743	1,20303	0,53375	0,76889	37,36121	34,21383	36,18772	37,14206

PS05-durin	PS05-after	05BSAverage	05PSAvera	1BSAverag	1PSAverag	1VASbefor	1VASdurin	1VASafter
31,58646	32,77867	1,56359333	32,51021	0,804977	33,14179	22	4	11
44,44448	44,93039	0,64704667	45,42183	0,74417	43,80983	0	7	0
38,88507	39,68783	0,40212667	39,29853	0,591287	39,84207	1	8	11
41,89583	40,92629	0,8249	41,40692	0,983953	43,88365	0	17	0
34,83763	36,20473	1,00536	35,87811	2,099103	39,37664	23	1	4
30,04102	30,67095	0,3277	30,25221	0,45336	30,9824	0	0	4
37,71666	38,08206	0,77399	38,51209	1,258483	40,03541	4	13	0
40,86111	41,76766	0,47672667	41,20725	0,743157	41,00078	0	0	0
34,47428	37,34316	1,66193	36,40185	2,54533	37,27961	1	0	0
28,02599	28,64214	0,3401	28,92802	0,35221	29,89703	0	10	1
36,83698	38,43801	0,94202	38,32167	1,443627	38,56618	0	3	1
32,13174	31,85202	1,25848667	32,15083	1,054773	32,62783	3	20	25
27,49427	27,82221	0,61168333	27,75614	0,882687	28,15957	2	2	3
28,85152	28,68048	1,45420667	29,72799	2,481613	30,40603	2	1	0
26,26609	28,21727	0,62951	27,58698	1,100837	31,18638	12	17	23
33,94265	35,92563	0,83522333	35,67011	1,274317	35,92092	2	1	1

05VASbefc	05VASduri	05VASafte	HIHzBefore	HIAmpBefc	HIHzDuring	HIAmpDur	HIHzAfter	HIAmpAfte	LIHzBefore
5	0	0	0,9	1,524016	0,75	1,280751	0,7	1,698901	0,9
0	2	0	0,9	2,013969	0,9	1,221577	0,3	2,165123	0,65
1	6	8	0,5	1,926303	0,5	1,468767	0,9	1,657247	0,85
0	0	0	0,75	2,488101	0,75	2,211871	0,9	2,01254	0,75
10	0	3	0	0	1,1	1,26812	1	0,846965	0,75
0	0	5	1,05	1,441648	1,35	0,852593	0,95	1,930974	1,2
3	7	0	0,9	2,163125	0,6	1,519678	0,7	1,804155	0,85
0	0	0	0,75	1,657045	0,8	1,211812	0,75	2,363595	0,55
10	0	0	0,75	1,583535	0,8	1,187409	0,8	1,432763	0,6
0	21	0	0,45	1,05751	0,6	0,637998	0,7	0,759491	0,65
2	2	1	0,75	0,822507	0,85	0,7321	0,55	1,366473	0,85
0	10	1	1,3	0,826032	1,35	0,791502	0,9	0,935192	1,25
7	2	6	1,15	0,681095	1,35	0,602508	1,1	0,48873	1,35
0	0	1	1,05	1,12048	1,05	0,79243	0,95	0,963457	1,7
34	35	46	0,55	1,747449	0,35	1,41285	0,45	1,280694	0,85
9	0	6	0,6	2,123721	0,7	1,08403	0,75	1,275731	0,45

LIAmpBefo	LIHzDuring	LIAmpDuri	LIHzAfter	LIAmpAfter
1,708462	0,95	0,999633	1	1,142743
2,010773	0,8	1,347021	0,8	0,958179
2,514111	0,95	1,149557	0,35	2,702266
1,857469	0,3	2,355608	0,85	1,884899
0,949154	1,4	0,684897	1,1	0,76273
1,054936	0,9	0,751251	0,9	1,358078
1,386639	0,5	1,355099	0,85	1,242074
2,061175	0,75	1,385894	0,6	1,922932
1,557783	0,95	0,911175	0,65	1,504092
0,935028	1,15	0,447856	0,75	0,819721
0,986344	0,8	0,831576	0,9	1,045356
0,912516	1,2	0,816641	1,15	0,828472
0,714059	1,2	0,649275	1,4	0,555603
1,605957	1,65	0,791072	1,65	1,206256
0,633584	0,45	0,430767	0,7	0,647321
1,377846	0,8	0,792079	0,75	1,112633
