# Supplementary material for: Intensified visual clutter induces increased sympathetic signalling, poorer postural control, and faster torsional eye movements during visual rotation
Source: PLoS One. 2020 Jan 3;15(1):e0227370. doi: 10.1371/journal.pone.0227370 (PMC6941927; doi:10.1371/journal.pone.0227370)
Supplement: Attachment — Submitted filename: Point-by-Point response.docx [file pone.0227370.s002.docx]

Dear Editor of PLOSONE,

We would like to express our thanks for your thorough evaluation of our previous submission. The comments added very valuable context to the manuscript, and allowed us to revisit it with new perspective. We understand that due to the usage of the same dataset as presented in a previously rejected manuscript (PONE-D.19-09571), PLOSONE requires a point-by-point response based on the previous comments. We are naturally happy to comply, hoping that we can address the issues with the previous submission.

As stated in the cover letter, the manuscript has been substantially revised. As such, we have performed a linguistic overhaul and restructured much of the paper, replaced many figures, added additional statistical analysis, and consequently drawn new conclusions. For this reason it will be hard to pinpoint specific alterations to a specific line in the submissions. While we hope you will consider this manuscript to be a new submission, we hope we can mitigate your concerns by answering the comments from the previous revision. Our answer to each point can be found highlighted in blue below respective paragraph.

**Reviewer #1:**

The purpose of this study was to explore correlate eye movement as a result of the OKN response with center of pressure in order to identify a generative visual mechanism for visual-vestibular mismatch. There is a major flaw in the design of the experiment in that the majority of visual¬-vestibular mismatch studies have advocated for dynamic balance disturbances. Static sway does necessarily promote a sense of mismatch. In addition, there is little evidence that visual flow and changes in COP is directly correlated. The absence of significant results is not surprising here. The discussion makes assertions that exceed reliability of the results and there seems to be errors in the presentation of the figures.

Specific Comments

1. Although this study has some interesting results about torsional eye velocity during OKN, the presentation and conceptualization is very difficult to follow and needs thorough editing by a scientist who is a native English speaker. Some examples just in the first few pages:

P1, line 15: Be careful of jargon, e.g., “visual balance”. You are provoking the balance system through visual disturbances. How does that differ from the term “Postural balance” used on p2, line 40?

Line 42: Is Balance discomfort the same as instability? Reference or explain this term

Line 45: What is the “mismatch model” – reference or describe

Vestibular eye movements: Are you referring to the OKN?

Need to Reference terms from the literature (e.g., line 46)

Line 59: the VOR is reflective of head position and acceleration - not body posture. Spatial orientation is not reflected in the VOR, it is a perceptual process

2. Line 73-75 does not make sense: How does the visual scene rotate as a result of a head tilt – Do you mean the VOR is impaired and the performer odes not compensate for head motion? Is this visual stimulation as you claim or impaired feedback?

3. I suspect the study is underpowered with 4 subjects per group with age and gender as factors.

4. Why is figure 6 labeled pupil size when the legend describes it as body sway?

The linguistic concerns have been addressed in the new submission. We have also made the specific changes recommended above. As for the group-analysis concerning age and gender, we have repeated all analysis without these variables, instead describing the study as matched for age and gender. This new statistical analysis was done using Bayesian ANOVA, which provides valuable context through its Bayesian factor. Consequently, we hope to be more convincing in our new interpretation of the results.

**Reviewer #2:**

This study tested balance and eye movements in 16 healthy adults in response to a visual flow of oblique lines. The measurements are interesting and carry the potential for clinical innovation. My main concern is regarding the gap between the question (sensory processing in people with dizziness or visual sensitivity) – the study design& sample (healthy adults, short exposure)-results (minor differences with high intensity and some in time) and conclusions (an oculo-vestibular pathway exists). The paper is claiming to provide evidence that an occulo-vestibular pathway exists by showing certain eye movements in response to an oblique visual flow. However, ocular torsion and vertical skewing are normal eye movements as a result of the ocular muscles action in response to a specific visual stimulus regardless of any vestibular component or pathway.

We agree that the conclusion of the previous submission was beyond its scope. We have therefore interpreted the results, focusing on describing the response in relation to the clinical assessment of vertiginous patients, not on the neuroanatomical aspects of the findings. We have also adjusted the title to be more in line with this new approach.

The participants had no motion sickness, nor was there a comparison to people with complete vestibular loss, for example to show differences between normal ocular movements and movements with lack of vestibular input. The head was steady as well (comparing to head tilts could also demonstrate vestibular connections). Additional major concerns about the paper are multiple statistical tests and overall lack of clarity. The scale on all figures should start at 0 and standard errors should align (rather than one top and one bottom – or better yet, use boxplots). The current visually display magnifies small differences. Statistical significance is over-stated with small magnitude of differences and low to moderate correlations.

Our intention with this study was to focus on the response and variation among healthy individuals, not to include patients. Patients suffering from visual vertigo will be the focus in the next study, we plan to collect data on patients with vestibular and non-vestibular vertigo during autumn 2019 with the same protocol as described here. We understand that the skewed distribution of the data set was unconvincing. We therefore repeated all analysis, using JASP and Bayesian ANOVA, performing only one repeated analysis for all variables together so as to minimize the risk of type 1 errors. The Bayesian factor adds valuable context to the validity of the ANOVA results. Concerning the figures, the majority of them have been replaced.

Below are a few specific suggestions to enhance clarity and readability:

Abstract

Line 16: What do you mean by ‘typically’ here

Line 17: write out a number at the beginning of a sentence

The abstract overall is a little unclear. Strengthen the motivation connection (first and second sentence don’t relate). What was the projection device? mention that low and high was randomized if you report a related result. The conclusion over-reaching given that your sample included healthy adults with no dizziness. Oculo-vestibular pathway is your explanatory hypothesis based on your findings not an unequivocal conclusion.

Introduction

Line 63: typical

Line 100: “in response to” or “within” cluttered…

Line 102: shedding light on NORMAL physiological integration…

Line 104: define intense, define prolonged

Line 111: with healthy adults (not ‘on’)

Line 112: Throughout the manuscript you use ‘discomfort’ to describe a balance problem. This word is not typically used with respect to

balance. Perhaps replace with dysfunction / impairment?

Methods

Lines 125-127: I don’t think the sentences about subjective measurements of balance are necessary here. Same for the sentence at line 129.

The linguistic issues above have all been addressed. They may however not remain in the new submission, as several paragraphs have been removed or replaced.

Good explanation of the visual stimulation. I’m wondering if an actual screenshot replace (or added to) the drawing in Figure 1?

Line 176: you tracked head position in order to control for ‘no head movements. But the head was not fixed, right? Was there a threshold for what you considered ‘no movement’? did you repeat a trial / exclude participants based on a certain amount of head movement? This is a little unclear.

The issues concerning head position have been thoroughly developed in the new submission, and the head movement has now been illustrated in a plotted graph (figure 3).

Line 181: you mention average sampling rate of the WBB, how much variation does the sampling rate had? How did you get to 99.1 Hz?

The literature varies here with some studies reporting 30 Hz, 60Hz and a few had 100Hz. Inconsistent sampling rate is a known problem of the WBB and should be mentioned in the limitations.

The frequency has been calculated as an average from several recordings, all of which yielded the same frequency of 99.1 Hz. As you mention, 100 Hz seems to be the higher sampling rate of the WBB and our average is slightly below this. We have addressed this issue as a limitation in the Discussion. We also provide an explanation for how we attempted to mitigate any issues that might have been produced due to the inconsistent sampling rate (p20; r391-397).

Lines 187 -194: this part is completely unclear – how does having a separate control group viewing a blank visual scene eliminates proprioception as a confounder? It appears that it’s controlling for the visual manipulation (not proprioception) but then it’s unclear why it is a different group?

We have clarified why this control group was recruited, and provided additional context regarding the setup of the control experiment (p8; r154-161).

Line 195: since you used the term ‘discomfort’ to describe balance before, it is unclear what kind of discomfort you are referring to here.

What were the participants asked? What was the purpose of this question?

The exact formulation of the question has been provided in the manuscript, as has additional reasoning for its implementation (p8; r164-169).

Line 202: the main outcomes were

Line 205: remove and before size of pupil

These issues have been addressed.

Results

Line 242: You had 4 age groups and d you designed the stimulus direction so the sentence about no sig difference in age and stimulus direction is unclear. Did you analyze the sample as one group because there was no effect of age? If so, please explain

Age has been removed as a factor.

Line 243: the sentence about position of SD line should be moved into the appropriate figure caption.

The figures are well done (esp those of the raw data) and are very helpful to understanding the results.

Line 290: I think what we’re seeing is the changes only happened in AP sway (ML sway stayed fixed throughout)

Line 293: there was a significant decrease during the stimulation (compared to before). Was the pupil size significantly different after the stimulation as well? Qualify that these are small changes (albeit statistically significant).

Consider a separate title for the pupil comparisons (as you did for eye movements) for clarity?

Results overall – you present different analysis / description for every outcome even though the structure of figures is the same. To increase readability I would recommend keeping the presentation of results short and consistent between the various outcomes.

A majority of the figures have been replaced, and those remaining have been revised. The structure of the results section has been shifted to match the new results as provided by the new statistical analysis, and we have made an effort to address the pupil size under both Methods and Discussion as per recommendation.

With respect to correlations – please focus on the magnitude of the correlations rather than their statistical significance. Define weakmoderate- strong or poor-good-excellent based on the literature (e.g., Portney and Watkins?) and interpret them as such. R of 0.46 or 0.42 are similar R of 0.04 is a non-existing correlation regardless of P values (line 331). You present correlations of subjective discomfort but then have a separate section for correlations. Please reorganize the division for sections in the results.

All correlations have been revisited with this feedback in mind. We have subsequently also changed our interpretation of the results and our conclusions.

Discussion

Reword line 343 as a secondary aim?

Line 360: “in response to” (rather than just ‘to’)

These changes have been addressed. We have formulated the analysis of correlations between variables as a secondary aim.

With the hope that you may consider this manuscript as a new submission,

Best Regards,

Tobias Wibble
