# Supplementary material for: Intensified visual clutter induces increased sympathetic signalling, poorer postural control, and faster torsional eye movements during visual rotation
Source: PLoS One. 2020 Jan 3;15(1):e0227370. doi: 10.1371/journal.pone.0227370 (PMC6941927; doi:10.1371/journal.pone.0227370)
Supplement: Attachment — Submitted filename: Point_by_point_response.docx [file pone.0227370.s003.docx]

Dear Dr. Anson,

Thank you for the valuable suggestions provided in regards to our submission “Intensified visual clutter induces increased sympathetic signalling, poorer postural control, and faster torsional eye movements during visual rotation”. We have done substantial revision to our original manuscript on the basis of the reviewers’ comments. We present here a point-by-point response with regards to the original feedback, and what changes we have made. The comments are organized in relation to respective reviewer, with our answer highlighted in blue. Changes in the manuscript have also been highlighted in blue, with line-references added in this letter for your convenience. In some cases we have performed general revision, and as such cannot point to any specific point in the manuscript. These changes are also highlighted in blue so as to be easily identified.

Thank you for your feedback and we look forward to hearing from you regarding our revision,

Tobias Wibble

**General**

1. We note that you have indicated that data from this study are available upon request. PLOS only allows data to be available upon request if there are legal or ethical restrictions on sharing data publicly. For more information on unacceptable data access restrictions, please see

http://journals.plos.org/plosone/s/data-availability#loc-unacceptable-data-access-restrictions.

In your revised cover letter, please address the following prompts:

a) If there are ethical or legal restrictions on sharing a de-identified data set, please explain them in detail (e.g., data contain potentially sensitive information, data are owned by a third-party organization, etc.) and who has imposed them (e.g., an ethics committee). Please also provide contact information for a data access committee, ethics committee, or other institutional body to which data requests may be sent.

b) If there are no restrictions, please upload the minimal anonymized data set necessary to replicate your study findings as either Supporting Information files or to a stable, public repository and provide us with the relevant URLs, DOIs, or accession numbers. For a list of acceptable repositories, please see http://journals.plos.org/plosone/s/data-availability#loc-recommendedrepositories.

We will update your Data Availability statement on your behalf to reflect the information you provide.

We apologize for this oversight and are happy to provide the original data set which we will upload with the revised manuscript.

2. Please ensure that you refer to Figure 2 in your text as, if accepted, production will need this reference to link the reader to the figure.

This error has been corrected and figure 2 is now references in the text (line 305; line 316)

**Reviewer 1**

Specific comments:

Abstract:

Line 18: Describe difference in intensity as later (line 23) you mention specific results for high intensity but have not described what the difference is.

We have added the essential specifications of what constitutes low and high intensity (line 18-19).

Introduction:

Line 56: Define ocular balance-response, reference or define.

The ocular balance response refers to the physiological eye movement reflex to visual and vestibular stimuli. As it is currently not in widespread use we have however chosen to remove it for increased readability.

Line 58: Reword and describe “they are, however, also seen to rotational optokinetic stimulation.”

We have merged this sentence with the previous section for increased fluency. In addition, we have described the visual stimulation as a visual scene being tilted, producing an optokinetic response.

Line 73: Be wary of performing correlations between all variables. Ensure you have clear reason/hypotheses behind correlation performed.

We have added a justification and explanation (line 88-94).

Methods:

Line 80: Define or reference balance discomfort. Does this mean instability.

Any previous balance diagnosis, such as BPPV or vestibular neuritis, were grounds for exclusion, as was a history of any subjective feeling of balance problems. Such problems could have been perceptual, subjective dizziness, or actual instability. We have added more context to our initial phrasing (line 98-100).

Line 81: “Subjects were balance in terms …”. In a paper investigating balance, maybe use the term “subjects were age and sex matched” to avoid confusion.

We agree that the word could add unnecessary confusion. We have summarily changed our phrasing.

Line 81: “Subjects were balanced in terms of age and sex (4 men and 4 women aged 19 to 25, and

4 men and 4 women aged 49 to 65)”. Define the two groups and why do the ages not match even though you state they are?

There were 4 men and 4 women in the young age group, and 4 men and 4 women in the old age group. As postural control traditionally is decreased with age we chose to have recruit within these two groups. The effect of aged was analysed and no significant effect was found. It is well-established that pupil diameter increases with age, which meant that the baseline values were different in this study. This did however not affect the analysis performed in this study as it dealt with the within-subject changes over time, unrelated to baseline values. This has now been further detailed in the manuscript (line 100-102; line 296-300).

Line 86: Were all canals assessed using HIT or just horizontal canals?

All three canals were tested. This has been explained in the manuscript (line 106-107).

Line 93: Reword sentence

We have reworded the sentence.

Figure 1: Legend and figure do not match. “A represents the high intensity stimulus (LI)” but figure 1A is a high intensity stimuli

We have corrected this oversight.

Figure 1 legend: Correct “0,42cm”

This has been corrected.

Line 129: Provide further detail about calibration process

We have added further explanation on the calibration process (line 165-168).

Line 134: “control that the head was immobilized”. Was the head actually immobilized or were participants instructed to not move the head?

Participants were instructed to not move their heads, and this was controlled using the accelerometers. The explanation for this has been adapted for increased readability (line 113-118; line 171-172).

Line 137: What was the specific criteria for rejection “noticeable movement”

An acceleration of the head below 1 deg/s^2^ can generally be seen as below the vestibular threshold. We have added an explained on how it was ascertained that participants did not go above this level (line174-178).

Line 144: Why does the WBB sampling frequency vary? Do you analyze data using each trials specific sample frequency (i.e. for filtering purposes) or treat all trials as though they were sampled at 99.1 Hz

The dynamic sampling frequency is a feature of the WBB software, not allowing for a constant sampling rate. As we are only dealing with large sections of data we do not consider this to be a major obstacle in the interpretation of the results. Had we looked at the velocity or specific characteristics of the body-sway then a higher time specificity would have been necessary. As it stands now, looking at the area over a long period of time, the sampling difference of a few frames has negligible impact on the results, and would have readily fallen out of the 95% ellipse during the analysis. Consequently all trials were analysed as sampled at an average of 99.1 Hz.

We have added additional reasoning on the analysis of the WBB data including references to the validity of the methodology (line 247-253). We believe this expands on our section in the discussion on the use of a dynamic sample rate as a possible limitation.

Line 159: Why was a blank visual field used and not a stationary version used during baseline in other trials?

This was chosen because the stationary visual field resulted in different responses already during the stationary phase on both body sway and pupil size. So the control trials had to be done with a more neutral visual scene as it was the effect of the visual scene that was being controlled for. We have added additional context behind this choice in the manuscript (line 194-197; line 200-201).

Line 162: As discomfort was not defined, it becomes hard to interpret any results collected via VAS. Are participants reporting instability or motion sickness.

The instruction related to the feeling of discomfort was intentionally left vague. The premise of the study was to keep the trial procedure intact and only change the visual stimulation. In the same way that the objective variables were then attributed to this change, it can be inferred that any change in VAS discomfort would be due to the change in visual conditions.

Additionally the VAS scoring was to be related to objective measures such as pupil size, indicating the sympathetic response. This response would not take the source of the stress into account, meaning that the pupil size reflects general stress levels, making it impossible to distinguish whether it was from postural instability or a general reaction to the visual motion. This is why further correlations were made with the body-sway as well.

We would suggest that the general phrasing of “discomfort” therefore is preferable in this study. We are of course open to further discussion on the matter and further clarification. We have added additional context in the manuscript (line 210-215).

Line 196: ‘In the Origin software’?

The proper way of introducing a software has been used (line 255-256).

Line 221: ‘Alfa’

Bayesian statistics does not have an alpha as it does not produce a probability. Rather, it produces the likelihood that a null hypothesis should be rejected, working in tandem with the ANOVA analysis. The ANOVA alfa has previously been reported.

Results:

Line 233: “in the compensatory direction” describe what this compensation is (i.e. visual rotation clockwise evoked ……..)

We have now explained this reasoning further (line 302-304).

Table 1,2&3: Units

We have added the applicable units in the table heading.

Figure 2 is not referenced in text

Figure 2 is now referenced (line 305; line 316)

Figure 2&4: Left and right graphs could benefit from being label A and B to aid in-text referencing.

We agree that this would increase readability and the labelling has been added.

Figures 2,4&5: Units and what do error bars represent (STD or SEM).

The error bars presented are SD, and an explanation has been added in the figure. Figure 5 has no error bar, and so we kindly ask for additional clarification on what aspect is lacking.

Figure 5: correct y axis numbering (0.5 not 0,5 etc)

This has been corrected.

Line 300: units

We have since our last submission been in contact with the manufacturer to add further clarity into this matter. The pupil size is measured in pixels. This naturally makes it difficult to estimate the actual diameter in SI units, but does allow for comparing the effects of the independent variables. This has been further commented on in the manuscript (line 222).

Line 315: Correlation figures could be beneficial

We would be happy to provide additional figures if it is deemed that it would increase the readability of the manuscript. As there are quite a few correlations made it would produce a rather large matrix-figure. We suggest that such a figure might take up a big part in an already rather long manuscript. Are there any specific correlations that is thought to be better displayed as figures rather than in-text notations?

Discussion:

Line 349-351: How??

We have added an explanation of how an increased frequency leads to increased retinal motion and eye movement in a torsional smooth pursuit.

Line 356: Reword sentence

The sentence has been shortened.

Line 363-364: If skewing is purely a vestibular response. Shouldn’t you see no response as all as the head is stationary, thus no vestibular input?

Correct, this referred to the previous notion that it was thought to only be a vestibular sign and thus can be expected to be less noticeable to visual stimulation. We have opted to remove this sentence as it only reiterates this sentiment from the introduction.

Line 420: Does balance discomfort actually mean balance impairment/ instability.

We interpret balance discomfort to be the purely subjective sensation of imbalance, which in itself is problematic for many elderly. It is however also correct that there is often objective instability. We have updated the nomenclature to reflect this.

Line 430: ‘even when the visual scene was stationary’ The visual scene was not stationary, or are you referring to the blank scene for the control group.

The visual scene was stationary for 10 seconds before and after each active rotation of the visual field, as described in the method section. The pupil size and body sway were significantly different during these initial 10 seconds between subjects viewing the low intensity stimulus and the high intensity. We have added some extra text to reflect this.

Line 431: reword

Has been reworded for clarity.

Line 440: I find it hard to understand this section as subjective discomfort is not defined, so unsure as to what participants were trying to portray through their answers. You use the word discomfort to describe postural instability in this manuscript and then in this section. I think it would be best to not use the word discomfort in the context of postural instability, thus potentially removing some confusion.

We believe this comment to be in line with the one bringing up the ambiguity of “discomfort” above. Please see the provided answer as we believe it to cover also this comment.

Line 462: As previously mentioned, correlations should be performed to examine hypothesis and relationships. It would appear that you performed correlation without clear reason ‘ as there is no clear reason for why this correlation would be seen’.

The result was in contrast to the hypothesis, the inverse of which lacks any known reasons behind it, which is why there is no reason for this particular correlation to be seen. We have added additional context to our hypothesis and the reasoning behind the correlations in the introduction and methods (line 88-94; line 119-122; line 282-282; line 538-540; line 546-551).

**Reviewer 2**

This is certainly an important research topic and the manuscript is easy to read. However, I think the manuscript should be improved by clarifying the rationale for particular measurements taken and analyses performed (i.e., comparing ‘early’ vs ‘middle’ vs ‘late’ for some measures and before and during rotations for others) in the study. In addition, some important information is missing in the methods, such as the task or procedure.

In the introduction, it would be helpful if the authors could provide more background information from the literature about why the particular measurements were chosen.

We have added additional reasoning for the sectioning of the eye movement response, as well as for our correlations between measurements. We believe this matter was also brought up by reviewer one and we would like to refer to the answer provided under the respective comments.

First, ocular torsion and vertical skewing are introduced as part of VOR, however the current study used visual rotation instead of vestibular stimulations such as head/body rotation. What is the relationship between visually-induced ocular torsion and vestibular ocular torsion? Is the relationship expected from the mismatch model?

The relation in terms of gain has been further elaborated on (line 60-65). The difference is not related to the impact of the mismatch model but rather on one being a vestibular response and the other a type of torsional smooth pursuit.

Second, body-sway and pupil responses were not introduced but only mentioned as other measurements. It would be helpful to provide more information about why measuring them, and how they would fit in the mismatch model. I’m not an expert in this field, but one relevant reference that comes to mind is, Lubeck, Bos, & Stins (2015), who showed that visual density of a rotation display affected body-sway. The paper by van Ombergen et al (2016) might also be relevant, as it describes the effect of optokinetic stimulation in vestibular mismatch patients (although they don’t assess eye movements).

We appreciate this valuable feedback and the suggestions on references, both of which were highly relevant to this study. We have consequently included both references and added additional reasoning surrounding the correlations (line 66-82).

Several important details and the rationale for performing the particular analyses are missing in the methods.

First, the procedure or task is not described. What were the instructions given to participants, especially regarding what they should do during the test? Were they sitting down or standing?

Was fixation explicitly required?

The rationale is introduced in the introduction now (see previous comments).

We have added a short introduction to the method section where we describe the key concepts. By choosing this approach over adding additional information beneath each subtitle we hope the clarity and readability is improved (line 113-121).

Second, it is not clear why the statistical analyses were performed in this manner. The authors describe that the aim is to investigate the effect of rotating visual clutter (line 68), and the hypothesis is that higher intensity would result in stronger responses (line 74-76). Following this, I would expect the authors to compare responses during static and rotation stimuli, with intensity as the factor for ANOVA.

Static and rotation stimuli were compared for pupil size and body sway, but not eye movement response as no such activity is expected during a static visual presentation. Consequently, any correlation between the eye movement response and the pupil size or body sway was done only during the active motion. The eye movement response was consequently an average of the Early, Middle, Late responses put together, and then compared to the other variables. This was poorly explained and has been added under the method section (line 194-197; 200-201).

It is not clear why the authors chose to use ‘Early, Middle and Late’ as time factors, and why eye movement responses and body-sway/pupil responses were analysed differently. It is also unclear whether the correlational analyses were corrected for multiple comparisons.

From our previous study we knew the ratio (torsion:skewing) changed, so we wanted to know if torsion or skewing changed the most. By dividing into time zones we could see how the two eye movement reflexes differ in response to visual stimulation. Skewing reaches a plateau while torsion dies out gradually. The average response is used for correlations between body sway and pupil size, but the three phased response is to discern how torsion and skewing behave to visual stimulation, and which one is responsible for the shift in ratio and in what way. Torsion is clearly more adaptable.

This was poorly explained and has now been in included in the analysis section (line 231-239).

It now also related better to the discussion where torsion and skewing is compared (line 416-418).

Some minor comments, mostly regarding clarification of procedure and analyses, or further discussion about interpretation are:

1. Line 81. It is hard to ignore the age gap between participants, which intuitively would have an effect on vestibular functions. Age should be considered as a factor in the analysis. If not, maybe it would be helpful to just use a few sentences to explain why.

We agree that this is an important aspect that should have been explained further. We have performed analyses with age as both an independent variable and a covariate, with no significant results relating to the change in dependent variables. The pupil size was significantly smaller at baseline in the older age group, which is well-known as pupil size decreases with age. This did however not impact the size-change within-subjects over time. This has been updated and given additional context (line 100-102; line 296-300).

2. Line 93. “The visual stimuli showed xxx”, should be “The visual stimuli are/is xxx”.

This has been adjusted.

3. Line 100. What do the authors mean by ‘evenly randomized’? Was one participant exposed to one direction only?

Participants were exposed to only one direction but both visual intensities. It was the order of presentation, i.e. the intensity of the first stimulus, which was balanced between participants. An explanation has been added.

4. Line 117. “0,42cm” should be “0.42 cm”.

This has been adjusted.

5. Line 131. Were the 3D head movements also recorded by Chronos, or a different system was used? What was the system used?

It was the same Chronos system, which has now been stated in the line.

6. Line 148. It is not entirely clear to me what the raw data from the WBB consists of, and how the authors processed these data. Also, given the differences in A-P and M-L body sway, the authors might want to consider treating these as separate variables. I would expect that the stimulus should only affect M-L sway, and the A-P sway is unrelated to the stimulus.

Using an ellipse to cover the shift in CoP is to our understanding the most common method of calculating postural instability, for which we have now included a reference. Consequently both ML and AP sway needs to be considered for it to form an elliptical pattern. Furthermore, if the AP sway is unrelated to the stimulus then that should have been revealed in the control experiments with participants viewing a neutral scene.

The explanation for the ellipse has been further developed under the analysis section (line 247-253).

7. Line 154. Proprioception and muscular or joint fatigue are not the same. Please clarify the rationale, preferably with citations.

As the reason behind the fatigue is of less importance we have opted to use the term more generally, as it is any type of fatigue-related confounder we want to exclude. We have removed the use of the word “proprioception.” We have also included a reference dealing with fatigue under the standing conditions used in our study. As the main point is to exclude all non-visual confounders, the purpose of the control experiments was to keep the conditions from the proper trials but instead present a neutral visual scene. Fatigue is the most likely confounder, but perhaps not the only one. We have now included this reasoning in the manuscript (line 194-197).

8. Line 174., Could the authors clarify how the raw data were processed? For example, what filters were used to process raw position data, and what criteria was used to separate quick phases and slow phases, and over which time intervals were each of the variables calculated?

This process was carried out in line with previous studies. The eye movement responses are plotted in a graph and visually inspected. The data was not filtered but extreme data points due to blinking, lost eye etc. were manually removed. The nystagmus beats can be very easily identified as can be seen in figure 3. The slow phase is the period between the nystagmus beats. The slope was calculated in the usual fashion of deducing velocities, i.e. distance divided by time. This has now been further clarified in the manuscript (line 228-231).

9. Line 187. Related to the major points described above, why were three time intervals chosen, and why are there ‘breaks’ between the time intervals?

This was brought up as a major comment above. Please see the provided answer.

Concerning the breaks, there were two reasons for implementing these. Firstly, the eye movement responses are highly individual, meaning that there is no way to predict if one section will have a higher or lower number of nystagmus beats. Creating sections therefore makes sure that at least more than one will be included in the analysis. Since the responses are so widely spread it would not be possible to create a ramp where each slow-phase is plotted against that specific time stamp. The breaks allowed a clear cut between the sections without any question on which section it should belong to, as a slow-phase does not respect the pre-set time boundaries. Secondly, the manual plotting and analysing of these traces were very time consuming and weren’t considered sufficiently justified.

10. Line 191. It is not clear why the body sway area was calculated this way. Wouldn’t the vertical and horizontal positions produce a trajectory rather than a scatter plot? Is it common to fit an ellipse to define the body sway area?

We interpret this comment to deal with the same type of question brought up in comment 6. Please see the provided response.

11. Line 210. Should “between” should be “within” here?

This refers to the order of presentation of the two intensities. This has been clarified in the manuscript.

12. Line 211. Were any post-hoc tests performed? If yes, what type?

No post-hoc tests were performed.

13. Results. There is redundancy in the presentation of the data. Figures are more preferable than tables. Several tables are redundant, such as table 1 (contents already shown in figure 2) and table 3 (figure 4).

This is correct. Tables 1 and 3 have been removed.

14. Line 247. Statistic test values are missing. What test was performed here?

We are not quite sure which analysis is considered to be missing. We have provided the data from the ANOVA, and as the next test was insignificant we have not included it. We would therefore kindly ask for an elaboration on this comment.

15. Figure 2 is not mentioned in the text.

This has been corrected.

16. Figure 3. It would be helpful to show these raw data before the averaged data. There are some inconsistencies in the figure. For example, the y axis says “Amplitude (degrees)”, but one of the legends says “head velocity”.

A second Y-axis has been added to match the plotted lines. As for the raw data on head velocity we are happy to provide that plot (please see figure A in this document). Due to subjects maintaining head stability very well the plots on head velocity are primarily presenting baseline noise levels. We therefore suggest that the smoothed data may be more appropriate to present in the manuscript, as the noise levels make the small, albeit insignificant in terms of provoking a vestibular response, difficult to see.

Figure A: The raw head velocity data compared to the smoothed plot presented in the manuscript.

Line 329. The VAS shows a strong floor effect, and I therefore don’t think it is appropriate to include this measure in the correlation analysis.

We agree with this assessment. Our ambition with retaining the VAS score in the analysis was to add a subjective reference to the objective variables. However, as is correctly addressed by reviewer 2 there is little value in using the VAS in a healthy population who are all expected to present low scores.

We therefore agree that the VAS score be removed from the correlation analysis, instead being presented with reference to the descriptive statistics already included. The discussions relating to this will remain much the same considering the non-results of the correlation analysis. We have removed the related lines in the discussion and instead added more context on the floor effect. We appreciate this point of feedback as we feel it has improved the readability of the manuscript (Line 546-551).

17. Discussion. The authors should discuss the effects of stimulus intensity during the ‘before’ period. It would also be helpful to discuss the conflict between the hypothesis and results regarding body-sway, namely that body sway seems to decrease during rotation. Is this true for both A-P and M-L sway when these measures are analysed separately?

These measured were not analysed separately as we opted for the most common way of quantifying body sway. As we are not interested in directional body sway, but rather the general postural imbalance to a certain condition, we chose the more encompassing approach and have not treated A-P and M-L separately. As we illustrate in figure 5, and acknowledge in the discussion, it was predominantly the A-P sway that was affected. As such it was predominantly the A-P sway that was affected over time. We have added an additional note on this trend in the discussion (line 490-491).

We have also highlighted the importance of the Before-period finding in relation to our discussion on the impact of visual clutter (line 510-514).

18. Discussion. Pupil sizes can be affected by many factors, and one possible confound in the current study could be luminance. I don’t think luminance of five points on the screen (line 109-111) can represent the average luminance of the display, since the visual stimuli are not uniform across the space. If the contrast of the line and the background is the same in both intensity conditions (line 103), then the average luminance of LI is higher than that of HI, which potentially could lead to smaller pupil sizes. This could also explain why the difference in pupil size exists even when the visual stimuli are static. The claim that pupil size can be an indicator for torsion (line 473-474) is therefore not convincing. Could the authors clarify or discuss this?

The effect of luminance is naturally a tricky one, since it involves measuring a moving screen hitting the retina. This is why the current methodology was adopted since it entails an artificial fovea, in which you detect light levels at a given time. The five point measurements resulted in 15 measure points per trial as they were all repeated three times. It is true that one might expect a substandard projector would produce a higher candela for the LI than the HI since there was more white area projected. No such tendency was observed under any condition. For the stationary screen the candela went from a mean of 927.5 to 927.6 from HI to LI. For the moving stimulation it actually decreased from 939.9 to 908.3, albeit far from significant, p=0.6.

The HI stationary luminosity changed from 927.5 to 936.9 when moving. The LI went from 927.6 to 908.3, p=0.8 and 0.7 respectively. As such there was no tendency of the luminosity being involved as a confounder.

One could argue that the midpoint measure would be the best indication of the light conditions on the fovea. HI produces a luminosity of 1108 (sd 28.3) while LI was 1105 (sd 15.6) over all trials.

We considered presenting these values in the manuscript, but as it would produce a rather large table we thought it would take from the core messages. An alternative to using the artificial Fovea-measurement would be to calculate the average luminosity in the room. Considering that participants were focusing on one fixation point this does however seem like an inferior alternative.

We have added some additional context in the discussion as this presenting a potential limitation of the study (line 467-475).

19. Discussion. It is not clear what the authors mean by “work load” (line 370-371). If participants were just passively viewing the screen, what is the “work load” required? Could the authors provide some evidence that increased visual information results in greater work load?

A bigger movement, or a faster one, requires more energy to be carried out. An increase in torsional velocity therefore means that the brain’s signal to the eye muscle is increased. As work is denoted as Newton-meter, this means that a faster movement necessitates a higher Watt. This comment correctly points out that Watt is not the correct unit of workload, so we have altered the description to reflect this in the form of “work over time” (line 438-440). The evidence for this lies in the primary results of this paper, as visual information altered the muscle fibre activation through increased eye movement velocities.
