# Supplementary material for: Intensified visual clutter induces increased sympathetic signalling, poorer postural control, and faster torsional eye movements during visual rotation
Source: PLoS One. 2020 Jan 3;15(1):e0227370. doi: 10.1371/journal.pone.0227370 (PMC6941927; doi:10.1371/journal.pone.0227370)
Supplement: Attachment — Submitted filename: Point-to-point-response.docx [file pone.0227370.s004.docx]

Dear Dr. Anson,

Thank you for the valuable suggestions provided in regards to our submission “Intensified visual clutter induces increased sympathetic signalling, poorer postural control, and faster torsional eye movements during visual rotation”. We are pleased to hear that our first revision was well received, and we consider the additional comments provided to be well presented in relation to the purpose of the study. After additional discussions within the author group we have performed additional revision to our manuscript to further clarify important key points.

We present our point-by-point response with regards to the original feedback, and what changes we have made. The comments are organized in relation to respective reviewer, with our answer highlighted in blue. Changes in the manuscript have also been highlighted in blue, with line-references added in this letter for your convenience. In some cases we have performed general revision, and as such cannot point to any specific point in the manuscript. These changes are also highlighted in blue so as to be easily identified.

Thank you for your feedback and we look forward to hearing from you regarding our revision,

Tobias Wibble

Reviewer #2: I am pleased with the additions that the authors made in the introduction and methods, it very much improved the readability of the manuscript.

However, I still find the aims (in the introduction and discussion), hypothesis and corresponding data analysis somewhat messy. I think it is important to clearly define the aim, and formulate hypotheses and analyze the data in line with this aim.

The current (first) aim is:

“to investigate the effect of rotating visual clutter on a series of variables in healthy adults: 1) The oculomotor response of ocular torsion and vertical skewing, 2) the postural response, measured as body-sway, 3) the sympathetic response, evaluated through monitoring pupil size, and 4) the subjective sensation of discomfort, evaluated through a subjectively self-reported visual analogue scale (VAS).”

However, the authors then look at the effect of the density of the stimulus and at the time course of oculomotor responses during the viewing of rotating visual clutter. For body sway, pupillary response, and subjective discomfort, the authors look at the effect of the rotation and density of the stimulus (the hypothesis concerns the effect of density). I would mention the intensity in the aim and separately describe the aim for the oculomotor responses. Also, in the discussion (line

403-405) the authors write “It was found that the level of visual clutter contained in the visual scene had a strong impact on ocular torsion, body-sway and the pupillary response over time”.

However, the time course was only analyzed for torsion and skewing. Please be consistent in the description of aims, hypotheses, and analyses.

We have restructured the aims in the introduction to present a clearer flow of how the manuscript is to be interpreted. The density is now mentioned in relation to intensity levels, and the hypothesis is added after we discuss the effects of visual intensity, to make it clearer that this is one set of aims related to the density levels. We then bring up the aim of correlating the parameters, which we now believe to be better separated from the density levels discussed in aim one (line 85; 90-96). In the discussion we have removed “over time”, as this is indeed not the case for this particular analysis.

Furthermore, did the authors consider analyzing the full time course of the pupillary response (like torsion and skewing), since this is also a continuous variable? Finally, if there are differences between pupil sizes of younger and older people, this is a confound in the correlation analyses.

Scatter plots for the correlations would be a nice addition.

As it stands pupillary response was analysed the same way as body-sway in terms of being averaged over time. In a previous revision we discussed similar aspects of analysing and presenting the dynamic variations in body sway. In the same manner we made the choice to only average the data over the stimulation period, as well as before and after. This would certainly add to the finer analysis of how the pupillary response might change depending on different stages of the visual rotation, and its effect over time within the stimulation itself. The reason why we opted to refrain from this analysis in the end was that we wanted to present it in juxtaposition with body-sway, the COP-analysis of which would make it difficult to select finer time intervals. In the end we chose to present this aspect of the data as the average response to the visual stimulation and compare it to before and after periods, reflecting a broader response profile.

As for age, this was part of the reason why the group was balanced in terms of young and old, to counteract the effect of age. Additionally, the ANOVA controls for differences in baseline values as the outcome variable dealt with changes. We have added the statistical values associated with age in the introductory paragraph under results (line 298-301). We have discussed the addition of a scatter plot, and in this case suggest that such an addition might not contribute much to the readability of the article, as the result is non-significant and not necessarily related to the primary outcome of the study. We fear that such a figure might take focus from the other more relevant graphical illustrations.

I also have a few minor comments. Line numbers refer to the version in which the changes are highlighted.

Minor comments:

Previous comment 9: Why were three intervals chosen as opposed to, for example, two or four intervals? The choice seems arbitrary. If the authors are interested in examining the time course, why not analyze the full time course of the slow phase movements?

The choice can definitely be up to debate, and was initially made after a lengthy discussion. We could have chosen any number of time intervals or present the values in reference to time as a scaling variable. The initial decision to implement time periods rather than the full time course was taken for two primary reasons. As this aspect of the data was meant to be descriptive of the eye movement response over time we opted for presenting the results in a way that was easy to visualise. As figure 3, now changed to figure 2, illustrates there slow-phases can be quite long. Many other subjects instead showed a very high frequency of nystagmus beats, creating several slow-phases. Presenting each of these velocities on a scatter plot would certainly have been possible but one could argue that the discrepancies between individuals would produce a poorly distributed plot, even though the trend would have been much the same. We certainly see the advantages of such a plot, but would argue that as this aspect of the study does not deal with the particular eye movements over time, but rather their correlation to body-sway and pupillary response, we have opted for what we perceive to be a clearer visualisation. The second reason is related to the additional work of measuring the many minor slow-phases for certain subjects.

Through creating chunks of data we were able to collect clear and well-defined slow-phases within the same time frame, and make sure that no individual is overrepresented due to the amount of slow-phases exhibited as the data was averaged for each individual and time period. This naturally brings us to the other aspect of this comment, which is well justified: Why three time periods? Conceptually we felt that Early, Middle, and Late presented a nice chronology that was easy to get an overview of. This could certainly have been presented as only Early-Late, or in terms of quarters. One particular reason for choosing three periods relates to being able to keep a rather wide range. As stated earlier there was a discrepancy in the number of slow-phases exhibited between subjects. Had we referred to four or more time periods we would risk certain individuals having the same slow-phase for multiple segments, or some having only one clear slow-phase while others had several, which would make comparing the averaged responses between individuals problematic. Our current approach was therefore a middle-ground between these factors, and we are hopeful the current approach will be considered adequate as it stands. We are naturally open to suggestions and have had a dedicated discussion on the topic within the author group, which prompted this rather lengthy reply.

Previous comment 14: This comment refers to the sentences on line 247-250 of the original manuscript: “… there was no significant difference between the Middle and Late period, while the Early response was the highest. There was no significant effect of Intensity or Intensity order for the stimulation period.” Were t-tests performed for comparing Early, Middle and Late? Please provide the test values for these statistical tests.

We apologize for this oversight, and have now added the appropriate values. Rather than a t-test the values were retrieved through repeated contrast analyses between the time-periods for additional clarity. We have now provided the significance levels for both torsion and skewing (line 312-315; 325-328).

Previous comment 16: I’m sorry that this comment was unclear. I intended to suggest to present the raw data before the average data, that is, to swop figure 2 and 3.

This is a very sensible suggestion and we are happy to oblige. We have also changed the introduction to the figure to match the new order (line 308-311).

Line 66-67: What would be the use of a biomarker to identify vertigo? If the symptoms are present, do we need a biomarker?

This is a valid point, relating to a certain ambiguity in how broad the word may be interpreted. The ambition of the biomarker would naturally be to quantify these otherwise highly subjective symptoms. In that way ‘identify’ might be the wrong word as it on a more general level refers to the binary act of stating whether the patient has vertigo or not; as this is subjective no objective parameter would be able to contradict the patient’s statement. As point of clarification we have therefore changed it to ‘quantify’ to present our intentions further, which also ties it to the secondary aim.

Line 69-72: “We have … increased clutter.” This sentence is grammatically incorrect. Please correct to: “In a recent study, we have shown … per degree of skewing in response to increased clutter.”

This is an appreciated suggestion, and we have consequently changed the phrasing.

Line 78: A word is missing after “proxy”.

Yes, the missing word was “for”. We have adjusted this mistake.

Line 210-215: “Additionally … intensity level.” I don’t fully understand what the authors have written here, please clarify.

We agree that it was a bit convoluted. This dealt with our justification of not further clarifying the nature of any subjective discomfort. As the only variable that was changed between trials was the visual intensity, we conclude that any change in discomfort rating would be from this change. We have clarified this in the manuscript (line 212-215).

Figure 2: This is not critical, but it would be great if the authors could make a plot of the full time course of these variables during the 20 s of rotation for the LI and HI condition (i.e., select the slow phases and remove the quick phases, average across trials within each condition, then average across participants)

While we certainly appreciate the information such a graph would convey we have discussed a couple of issues related to presenting the information in such a way. These predominantly relate to the reasoning related to the previous comment on implementing intervals rather than a continuous timescale. As we understand it, the suggestion is to present all traces in one graph, with a highlighted average trace. Such a graph would convey very little information due to the nature of the torsional eye movements; the onset and duration of slow phases are very much uncoordinated between subjects. Each slow phase would therefore have to be adjusted for in respect to time, but since the number of interrupting nystagmus beats vary greatly within a healthy population it would not be possible to present all slow-phases in such a fashion. The resulting graph would be heavily cluttered with traces, and providing an average would not be feasible. An average could possibly be used to illustrate the initial slow-phase as it starts in synchronicity with the visual stimulation, but in this regard we would argue that figure 2 can be considered sufficient.

Nevertheless, we appreciate the input and hope our consideration is found reasonable.

Line 340: Please remove the sentence referring to table 3.

An oversight on our part, which now has been corrected.

Line 393-398: I apologize that I missed this in the previous round, but why is oscillation amplitude introduced here?

The variable is introduced earlier under analysis, line 253. It is also presented under the results section in line 368. It presents a more dynamic pupillary response than average pupil size, similar to the oscillation frequency. Naturally it is closely linked to the average pupil size, but we have included it as a pairing with the frequency. We have added a clarification in the manuscript on why this was done (line 254-256).

Line 438-449: I still don’t think the authors’ reasoning here is correct. Neurons can show tuning for visual spatial frequency or (rotational) speed but increased stimulation does not simply result in an increased signal, or increased ‘work’ for the brain. Please revise or provide references.

After further consideration we agree that this phrasing deviates from the appropriate nomenclature. Our ambition with this paragraph was to present the data in a way that might appeal to the clinical implications of the study. We have now rephrased it in a more descriptive way, suggesting that the correlation to visual vertigo may be through eye movement velocity rather than the inferred work by the eye or brain. We have referenced the increase in feedback errors in the visual system, often seen due to discrepancies between visual and eye velocities, as a possible approach to this issue. We find this to be of particular interest as it is sensory feedback errors that are attributed to most cases of vertigo, and it relates to the primary findings of the study relating eye movement velocities to body-sway (line 442-453).
